# Dual RNA-sequencing of Fusarium head blight resistance in winter wheat

**DOI:** 10.3389/fpls.2023.1299461

**Published:** 2024-01-04

**Authors:** Philip L. Walker, Mark F. Belmonte, Brent D. McCallum, Curt A. McCartney, Harpinder S. Randhawa, Maria A. Henriquez

**Affiliations:** ^1^Morden Research and Development Centre, Agriculture and Agri-Food Canada, Morden, MB, Canada; ^2^Department of Biological Sciences, University of Manitoba, Winnipeg, MB, Canada; ^3^Department of Plant Sciences, University of Manitoba, Winnipeg, MB, Canada; ^4^Lethbridge Research and Development Centre, Agriculture and Agri-Food Canada, Lethbridge, AB, Canada

**Keywords:** AC Emerson, AC Morley, dual RNA-sequencing, F. graminearum, FHB, T. aestivum, transcriptomics, resistance

## Abstract

Fusarium head blight (FHB) is a devastating fungal disease responsible for significant yield losses in wheat and other cereal crops across the globe. FHB infection of wheat spikes results in grain contamination with mycotoxins, reducing both grain quality and yield. Breeding strategies have resulted in the production of FHB-resistant cultivars, however, the underlying molecular mechanisms of resistance in the majority of these cultivars are still poorly understood. To improve our understanding of FHB-resistance, we performed a transcriptomic analysis of FHB-resistant AC Emerson, FHB-moderately resistant AC Morley, and FHB-susceptible CDC Falcon in response to *Fusarium graminearum*. Wheat spikelets located directly below the point of inoculation were collected at 7-days post inoculation (dpi), where dual RNA-sequencing was performed to explore differential expression patterns between wheat cultivars in addition to the challenging pathogen. Differential expression analysis revealed distinct defense responses within FHB-resistant cultivars including the enrichment of physical defense through the lignin biosynthesis pathway, and DON detoxification through the activity of UDP-glycosyltransferases. Nucleotide sequence variants were also identified broadly between these cultivars with several variants being identified within differentially expressed putative defense genes. Further, *F. graminearum* demonstrated differential expression of mycotoxin biosynthesis pathways during infection, leading to the identification of putative pathogenicity factors.

## Introduction

Wheat (*Triticum aestivum L.*) is a major global cereal crop, accounting for approximately 20% of human consumed calories worldwide (FAOSTAT, 2019). A significant threat to global wheat production is through pathogen attack, which is estimated to be responsible for 21.5% losses in wheat yield globally (Savary et al., 2019). *Fusarium graminearum* Schwabe is the dominant causative agent of one of the most economically important fungal diseases in wheat, Fusarium head blight (FHB). FHB results in grain contamination with trichothecene mycotoxins, such as the virulence factor deoxynivalenol (DON), which reduces grain quality and yield, while also being hazardous for human and animal consumption (Shin et al., 2014). Economic losses from FHB are expected to increase with rising temperatures and humidity levels due to climate change, likely resulting in more frequent and destructive FHB outbreaks (Shah et al., 2014; Hay et al., 2022). Current preventative measures involve an integrated approach, combining the use of fungicides, cultural practices and FHB-tolerant wheat cultivars to mitigate FHB disease pressure; however, these techniques are currently unable to provide complete FHB resistance (Torres et al., 2019). Further, while FHB-resistant wheat cultivars have been identified, these cultivars display poor agronomic traits, making the generation of elite cultivars challenging (Huang et al., 2018; Yi et al., 2018).

FHB infection initiates on spikelets during wheat anthesis, where the production of mycotoxins and effector molecules contribute to the infection progressing into the rachial node and subsequently progressing up and down the rachis to adjacent spikelets (Dweba et al., 2017; Rocher et al., 2022). While several mycotoxins contribute to FHB virulence, the most influential and well-studied is the trichothecene mycotoxin DON. Prior studies have identified key regulators of DON biosynthesis within the trichothecene cluster, where *tri5* and *tri6* mutants were unable to produce DON, leading to reduced virulence and host colonization (Audenaert et al., 2014; Wang et al., 2023). As DON plays a critical role in pathogenicity, DON detoxification has been identified in FHB-resistant species and cultivars, both through the activity of glutathione S-transferases like *Fhb7* from *Thinopyrum elongatum* (Guo et al., 2023; Wang et al., 2020), and through UDP-glycosyltransferase activity in *Brachypodium distachyon*, where DON is conjugated into deoxynivalenol-3-gluscoside (Pasquet et al., 2016; Gatti et al., 2018; He et al., 2020). DON detoxification through UDP-glycosyltransferase activity has also been linked to the *Fhb1* quantitative trait locus (QTL), which has shown the strongest and most stable FHB-resistance in wheat and is derived from the most well characterized FHB-resistant cultivar Sumai 3 (Gunupuru et al., 2017; Su et al., 2019). While DON detoxification appears to significantly contribute to a successful host response against FHB, FHB-resistance is a quantitative trait and therefore influenced by several QTLs (Zhang et al., 2021). Research has focused on the identification and confirmation of QTLs in FHB-resistant cultivars using genomic and transcriptomic approaches, with thus far over 500 QTLs associated with FHB-resistance being reported (Wang et al., 2020; Buerstmayr et al., 2021; Nilsen et al., 2021; Xu et al., 2020). Improving our understanding of the genes and enriched host defense pathways associated with and shared between FHB-resistant cultivars and QTLs are critical in improving our understanding FHB-resistance and generating elite FHB-resistant wheat cultivars.

The plant defense response includes the activation of both physical and chemical barriers to deter pathogen progression. Physical defense can occur through cuticle thickening, as well as cell wall lignification and modification which are essential processes in slowing FHB due to the suite of cell wall degrading enzymes released by *F. graminearum* upon infection (Kikot et al., 2009; Mandalà et al., 2021). Cell wall lignification has been of particular interest to researchers, as multiple FHB-resistance QTLs have identified genes associated with lignin biosynthesis, including the previously mentioned *Fhb1* (Soni et al., 2021). Chemical defense responses to pathogen attack include the release of phytoalexins to deter challenging pathogens, as well as reactive oxygen species (ROS) as part of a hypersensitive response at the site of infection to limit pathogen spread throughout the host (Khaledi et al., 2016; Polturak et al., 2022). As these ROS accumulate at the host-pathogen interface, both host and pathogen require antioxidant activity to scavenge ROS and limit oxidative damage through redox homeostasis (Khaledi et al., 2016). This ability to regulate oxidative stress is essential to the host in mounting a successful defense response, as well as to the challenging pathogen throughout infection (Fernando et al., 2019; Matić et al., 2021). Further, priming of this host ROS response through increased antioxidant activity can provide an advantage to the host prior to and upon pathogen attack, leading to reduced disease symptoms (Naguib, 2018; Fujita and Hasanuzzaman, 2022).

The study of gene expression at the host-pathogen interface is essential to understand factors contributing to both pathogenicity and the host defense response. Previous identification of FHB-resistant cultivars provides an opportunity to compare resistant and susceptible cultivars and to explore putative differences at the molecular level (Graf et al., 2013; Pan et al., 2018). Among previously identified resistant winter wheat cultivars are FHB-resistant AC Emerson and FHB-moderately resistant AC Morley (Tamburic-Ilincic et al., 2011; Graf et al., 2013). While AC Emerson and AC Morley demonstrate increased resistance to FHB, the underlying molecular mechanisms leading to this resistance have yet to be explored. Investigation of these mechanisms can not only identify novel defense genes and pathways contributing to FHB-resistance, but further identify shared genes and pathways across previously examined FHB-resistant cultivars and provide additional resources in building resistance to FHB.

To better understand the *T. aestivum – F. graminearum* interaction, we profiled FHB-resistant AC Emerson, FHB-moderately resistant AC Morley, and FHB-susceptible CDC Falcon winter wheat cultivars in response to *F. graminearum* at 7-days post inoculation (dpi). Dual RNA-sequencing revealed expression of shared and specific defense gene sets between resistant cultivars prior to, and in response to, *F. graminearum* infection, while also identifying differentially expressed genes associated with DON biosynthesis and redox homeostasis in *F. graminearum*. Further, nucleotide sequence variants were identified in differentially expressed genes involved in these pathways and processes. Taken together, these data provide insight into defense genes and pathways that influence FHB-resistance, as well as the pathogenicity factors that may slow FHB disease progression.

## Materials and methods

### Fungal culture and macroconidia preparation

A highly virulent 3-acetyldeoxynivalenol producing isolate of *F. graminearum* (HWW-15-33), obtained from the Henriquez Winter Wheat (HWW) collection of *Fusarium* isolates at Agriculture and Agri-Food Canada, Morden, Manitoba was used in this study. For conidia production, *F. graminearum* preserved at -80°C (filter paper Whatman No.1) was plated onto a petri dish (100 mm) containing Spezieller-Nährstoffar Agar (SNA), and incubated at 22°C for 10 days under a combination of fluorescent-UV lights (Nilsen et al., 2021).

### Plant materials

Plant materials used in this study included the hard red winter wheat cultivars AC Emerson (Graf et al., 2013), AC Morley (Tamburic-Ilincic et al., 2011) and CDC Falcon (Fowler, 1999). ‘AC Emerson’ is a hard red winter wheat, F_1_-derived doubled haploid (DH) cultivar produced from the cross McClintock/CDC Osprey, and is resistant to FHB. ‘AC Morley’ is a moderately resistant (MR) cultivar, derived from the cross FR227/17//IP16/20. ‘CDC Falcon’ is susceptible to FHB and derives from the cross Norstar/2/Vona//Abilene. The plant growing conditions were similar to Nilsen et al., 2021. At the growth stage where 50% of the spikelets has anther extrusion, single floret inoculation was performed with 10 µL of *F. graminearum* inoculum at a concentration of 5 x 10^4^ macroconidia/mL, and water (control) between the lemma and palea at the midpoint of the spike. The inoculated spikelet was marked using a marker pen. This was repeated for five individual wheat plants and three replicates for all cultivars.

### RNA sequencing and data processing

Based on significant differences in disease symptoms identified between FHB-resistant and FHB-susceptible cultivars, 7-dpi was the selected time-point to examine gene expression patterns by RNA-sequencing. The spikelet below the point of inoculation was isolated at 7-dpi for *F. graminearum* and water inoculated treatments for each cultivar. Five spikes were used per replicate, one spike per plant/pot. Each treatment was replicated three times. RNA extraction was performed from 60 sectioned rachis nodes and rachilla samples using Trizol Reagent (Ambion), according to the manufacturer’s instructions. DNase I (Invitrogen) treatment was applied to each sample following the manufacturer’s instructions. RNA yield and quality was assessed using the Agilent 2100 Bioanalyzer and the Agilent RNA 6000 Nano Kit (Agilent). The average RNA Integrity Number (RIN) value for all the samples was 8.4 out of 10. cDNA libraries were prepared by using the NEB rRNA-depleted stranded (plant) kit. Samples were sequenced at the McGill University and Genome Quebec Innovation Centre (Montreal, Canada) using the HiSeq2500 Illumina sequencer with 125-nucleotide paired-end reads.

Raw and processed RNA-sequencing reads were deposited at the Gene Expression Omnibus (GSE233409). Adapter sequences and low-quality reads were removed using Trimmomatic v0.36 (Bolger et al., 2014) (LEADING:3 TRAILING:3 SLIDINGWINDOW:4:20 MINLEN:36) and surviving reads aligned to the *T. aestivum* (IWGSC-56) and *F. graminearum* PH-1 (ASM24013v3) reference assemblies using HISAT2 (Kim et al., 2015). Count data was generated using featureCounts (Liao et al., 2014) and used as input for clustering and differential expression analysis through DESeq2 (Love et al., 2014). Hierarchical clustering (**Supplementary Figure 1**) and principle component analysis (**Supplementary Figure 2**) clustering was performed using pvclust (Suzuki and Shimodaira, 2006) and DESeq2 respectively. TMM expression values were calculated using EdgeR (Chen et al., 2016). Differentially expressed genes were identified using an adjusted p-value cut-off of p ≤ 0.01 and log_2_ FC < -2, > 2 and sorted using Venny 2.1 (Oliveros, 2007). Subsequently, GO enrichment of identified differentially expressed genes (DEGs) was performed using ShinyGO (Ge et al., 2020) and visualized using the conditional formatting function in Excel. DEG lists can be found in **Dataset S1**. Variants were identified for each sample using BCFtools software (Li, 2011). Low quality SNPs were filtered using the BCFtools filter function based on quality (<20), depth (<10) and variant distance bias (<0.001). GO enrichment was also performed on coding sequences containing nucleotide sequence variants using ShinyGO (Ge et al., 2020).

### Quantitative real-time PCR validation

cDNA was generated using Quantabio qScript cDNA Supermix (Beverly, MA, USA). *T. aestivum* and *F. graminearum* RT-qPCR primers were designed using Primer3 and validated using the Primer BLAST tool. Transcript abundance was measured on the Bio-Rad CFX96 Connect Real-Time system using SsoFast EvaGreen Supermix (Bio-rad Laboratories, Hercules, CA, US) in 10 µl reactions according to manufacturer’s protocol using the following conditions: 95°C for 30s, and 45 cycles of: 95°C for 2s and 60°C for 5s. Melt curves were performed at a range of 65 – 95°C with 0.5°C increments to assess nonspecific amplification and potential primer dimers. Relative transcript abundance was calculated using the ΔΔCt method, relative to heterogenous nuclear ribonucleoprotein Q (hnRNP Q) for *T. aestivum* and β-tubulin (FGRAMPH1_01G26865) (Liang et al., 2023) for *F. graminearum* using three biological replicates per treatment and three technical replicates per biological replicate. RT-qPCR primers used in this study can be found in **Dataset S2**. RT-qPCR validation of RNA-sequencing data in both *T. aestivum* and *F. graminearum* can be found in **Supplementary Figure 3**.

## Results

### *F. graminearum* infection in AC Emerson, AC Morley and CDC Falcon

**Figure 1:**
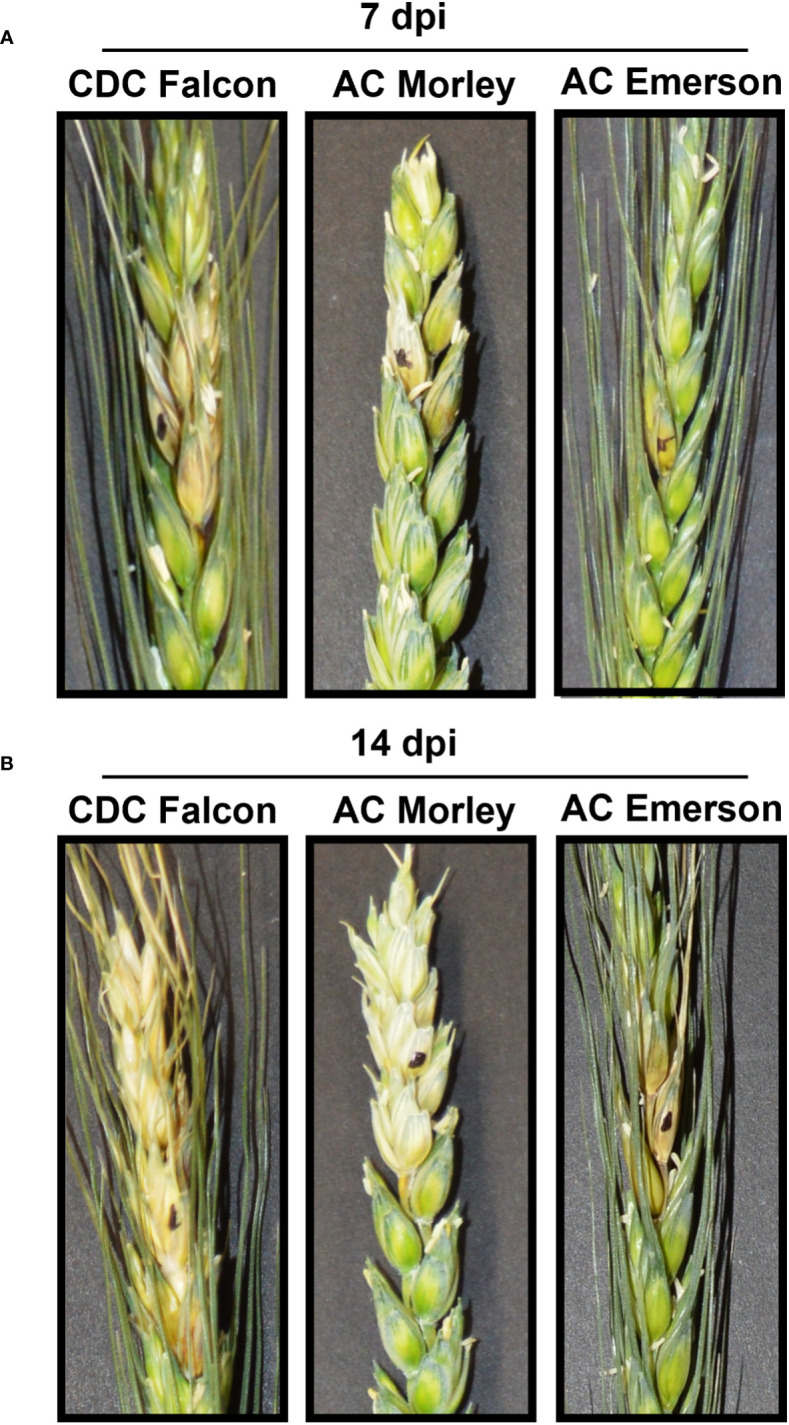
Fusarium head blight (FHB) disease severity in CDC Falcon, AC Morley and AC Emerson. Wheat spikes at **(A)** 7- and **(B)** 14-days post inoculation (dpi) inoculated with *F. graminearum.* Single florets were inoculated at 50% anther extrusion with 10 µL of 5x10^4^ macroconidia/mL.

Visual assessments of *F. graminearum* infected wheat spikes were performed at 7- and 14-dpi and used to score FHB severity in AC Emerson, AC Morley and CDC Falcon cultivars (**Figures 1A, B**). Here, FHB-susceptible CDC Falcon demonstrated the highest level of FHB severity at both timepoints with an average severity of 20.3% and 74.5% at 7- and 14-dpi respectively, where disease symptoms progressed into adjacent wheat spikelets as early as 7-dpi (**Figure 1A**)(**Dataset S3**). FHB-resistant AC Emerson demonstrated the lowest level of FHB severity (7.8% and 24.4%) with infection being limited to the point of inoculation at 7-dpi prior to progression down the rachis internode at 14-dpi (**Figures 1A, B**)(**Dataset S3**). AC Morley also demonstrated reduced FHB infection compared to FHB-susceptible CDC Falcon, with infection being limited to the inoculated spikelet at 7-dpi before progressing into adjacent spikelets at 14-dpi (**Figures 1A, B**). The 7-dpi timepoint was selected for dual RNA-sequencing to represent infected samples, being the earliest timepoint measured demonstrating significant differences in FHB severity between cultivars, while also providing sufficient time for *F. graminearum* to well colonize the FHB-resistant cultivar AC Emerson.

### Differential gene expression analysis of uninfected tissue reveals defense priming in FHB-resistant cultivars

**Figure 2:**
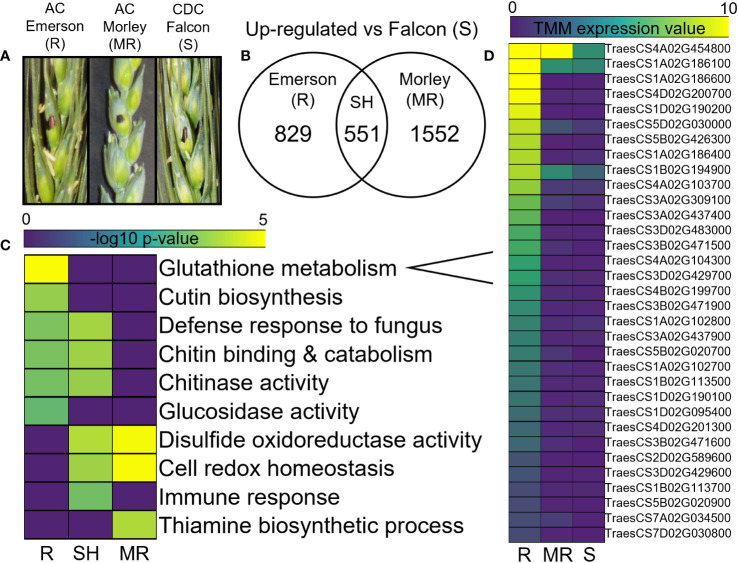
Differential expression analysis of AC Emerson (R), AC Morley (MR) and CDC Falcon (S) *T. aestivum.* **(A)** Control water inoculated spikelets for CDC Falcon, AC Morley and AC Emerson *T. aestivum* cultivars. **(B)** Significantly up-regulated genes in AC Emerson and AC Morley against FHB-susceptible CDC Falcon detected using DESeq2. **(C)** Gene ontology (GO) term enrichment of significantly up-regulated gene sets. **(D)** Trimmed mean of M-values (TMM) gene expression heatmap of glutathione S-transferases in *T. aestivum* quantified using edgeR. DEGs identified using DESeq2 with a log_2_ FC < -2, > 2 and an adjusted p-value cut-off of *p* < 0.01. GO terms considered significantly enriched at *p* < 0.05 prior to log_10_ transformation. R = FHB-resistant AC Emerson, MR = FHB-moderately resistant AC Morley, S = FHB-susceptible CDC Falcon, SH = shared. Yellow colour = more statistically enriched, purple colour = less statistically enriched.

Differential gene expression analysis of uninfected control tissues, compared between cultivars, identified both shared and specific significantly up-regulated gene sets in the resistant AC Emerson and moderately resistant AC Morley cultivars relative to the FHB-susceptible CDC Falcon cultivar (**Figure 2**). Here, 1552 and 829 genes were significantly up-regulated in AC Emerson (R) and AC Morley (MR) cultivars respectively, while 551 genes were significantly up-regulated in both resistant cultivars compared to CDC Falcon (S) (**Figure 2B**). Gene ontology (GO) term enrichment analysis revealed significant enrichment of oxidoreductase activity (*p* = 5.8x10^-4^), cellular redox homeostasis (*p* = 1.9x10^-3^), and the immune response (*p* = 2.0x10^-2^) in the shared gene set (**Figure 2C**). Further, we identified 33 putative glutathione S-transferases (GSTs), belonging to the glutathione metabolism (*p* = 1.4x10^-5^) enriched GO term, which were up-regulated in either the shared AC Emerson and AC Morley gene set, or the AC Emerson-specific gene set prior to infection (**Figure 2D**), suggesting priming of redox homeostasis in both resistant cultivars, with GST antioxidant activity playing an important role in AC Emerson. Further, Glutaredoxins (*TRAESCS5A02G094700, TRAESCS5A02G094900, TRAESCS5B02G100900, TRAESCS5B02G101100, TRAESCS5D02G107500*) and thioredoxins (*TRAESCS1B02G462300, TRAESCS6A02G323100*) involved in redox homeostasis were also enriched in our resistant cultivars prior to infection (**Dataset S1**), and like GSTs, have demonstrated enrichment in previous FHB-resistant wheat cultivars in response to FHB, playing an important role in the response to oxidative stress (Li, 2014; Kumar et al., 2020). GO analysis of the AC Emerson-specific gene set also revealed enrichment of cutin biosynthesis (*p* = 3.0x10^-3^), as well as chitinase (*p* = 1.6x10^-2^) and glucosidase activity (*p* = 4.2x10^-2^) (**Figure 2C**).

### Distinct defense processes are enriched in FHB-resistant cultivars in response to *F. graminearum*

**Figure 3:**
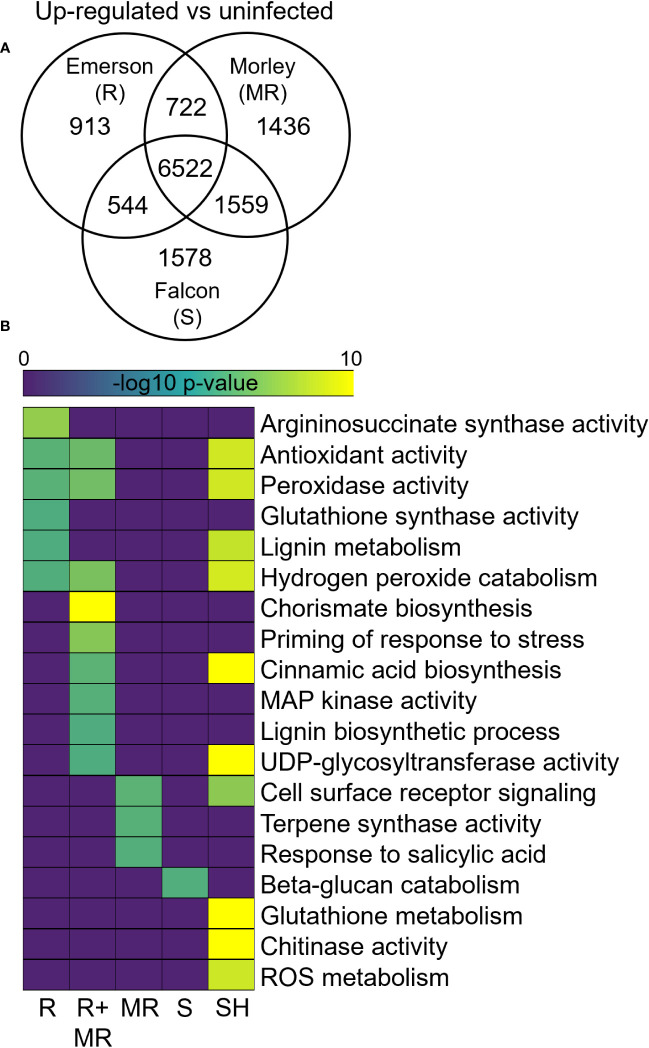
Differential expression analysis of AC Emerson, AC Morley and CDC Falcon *T. aestivum* at 7-dpi with *F. graminearum.* Gene ontology enrichment of significantly **(A)** up-regulated and **(B)** down-regulated genes in *T. aestivum*. DEGs identified using DESeq2 with a log_2_ FC < -2, > 2 and an adjusted p-value cut-off of *p* < 0.01. GO terms considered significantly enriched at *p* < 0.05. R = FHB-resistant AC Emerson, MR = FHB-moderately resistant AC Morley, S = FHB-susceptible CDC Falcon, R+MR = AC Emerson and AC Morley, SH = shared. Yellow colour = more statistically enriched, purple colour = less statistically enriched.

**Figure 4:**
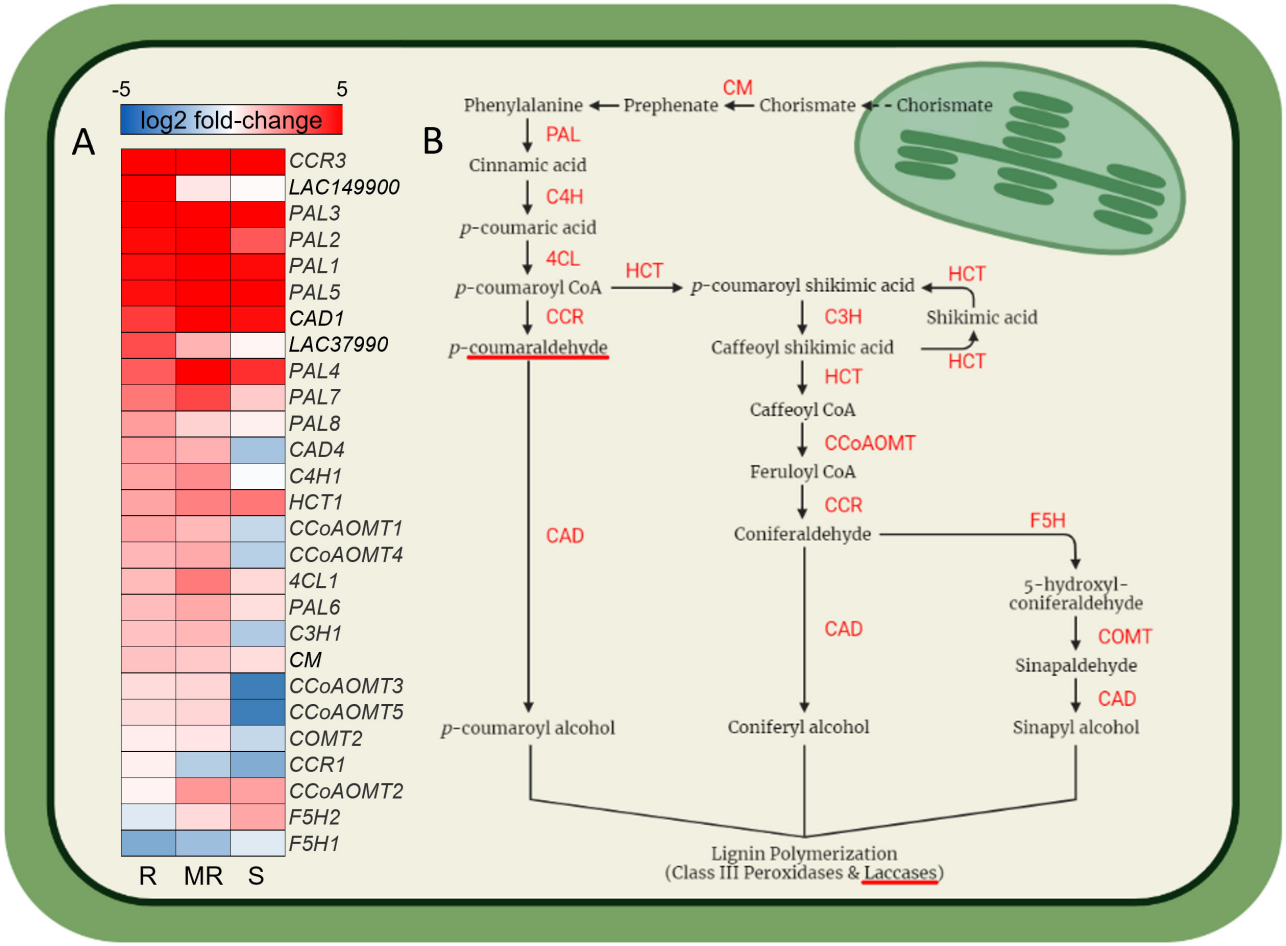
Gene expression heatmap of the lignin biosynthesis pathway. **(A)** Heatmap represents log2 fold-change between *F. graminearum* infected and control tissues for AC Emerson (R), AC Morley (MR) and CDC Falcon (S) cultivars. Log2 fold-change quantified using DESeq2. **(B)** Lignin biosynthesis pathway. CM, chorismite mutase; PAL, phenylalanine ammonia-lyase; C4H, cinnamate 4-hydroxylase; 4CL, 4-coumarate-CoA ligase; CCR, cinnamoyl-CoA reductase; CAD, cinnamyl alcohol dehydrogenase; HCT, *p*-hydroxycinnamoyl-CoA:qunate/shikimate p-hydroxycinnamoyltransferase; C3H, *p*-coumarate 3-hydroxylase; CCoAOMT, caffeoyl-CoA O-methyltransferase; CCR, cinnamoyl-CoA reductase; F5H, ferulate 5-hydroxylase; COMT, caffeic acid O-methyltransferase. Red colour = up-regulated infected tissue relative to uninfected, blue colour = down-regulation in infected tissue relative to uninfected.

Differential gene expression analysis of winter wheat cultivars in response to *F. graminearum* at 7-dpi compared to respective water inoculated controls identified both shared and specific up-regulated gene sets across AC Emerson, AC Morley and CDC Falcon (**Figure 3A**). For example, 913, 1436 and 1578 genes were significantly up-regulated specifically in AC Emerson (R), AC Morley (MR) and CDC Falcon (S) cultivars respectively, while 722 genes were up-regulated in both AC Morley and AC Emerson cultivars, and 6522 genes were significantly up-regulated across all three cultivars, compared to the water inoculated controls (**Figure 3A**). GO enrichment of these gene sets revealed distinct defense responses between cultivars, specifically between FHB-resistant and susceptible cultivars (**Figure 3B**). For example, enrichment of GO terms associated with physical defense like chorismate (*p* = 4.1x10^-9^), cinnamic acid (*p* = 1.4x10^-2^) and lignin (*p* = 4.3x10^-2^) biosynthesis, as well as DON detoxification through UDP-glycosyltransferase activity (*p* = 4.8x10^-2^) in the AC Emerson and AC Morley shared gene set (**Figure 3B**). To further explore the role of lignin biosynthesis in FHB-resistance, we mapped and plotted the expression of genes within the lignin biosynthesis pathway in response to FHB (**Figures 4A, B**). Here, we identified enriched mRNA levels of several key regulatory enzymes, including multiple caffeoyl-CoA O-methyltransferases (CCoAOMTs) associated with G-unit monolignol synthesis, shared between AC Emerson and AC Morley cultivars, while also identifying two putative laccases (*TRAESCS5B02G149900* (*LAC149900*) and *TRAESCS3B02G37990* (*LAC37990*)) that were specific to AC Emerson (**Figure 4A**). Interestingly, we also identified two ferulate 5-hydroxylases (*F5H1* and *F5H2*) involved in S-unit monolignol synthesis that showed down-regulation in resistant cultivars relative to CDC Falcon, suggesting lignin composition may differ between our FHB-resistant and susceptible cultivars (**Figure 4A**). Fold-change of UDP-glycosyltransferases involved in DON detoxification was also plotted in response to FHB and demonstrated significant enrichment in both AC Emerson and AC Morley cultivars, however, AC Emerson showed a stronger up-regulation of these genes in response to infection (**Supplementary Figure 4**). Specifically, four UDP-glycosyltransferases demonstrated extremely high levels of up-regulation between both resistant cultivars (*TRAESCS2B02G088400* and *TRAESCS2A02G028000*) or specific to the AC Emerson cultivar (*TRAESCS2A02G056400* and *TRAESCS2B02G626500*) (**Supplementary Figure 4**). GO analysis further identified enrichment of processes associated with the oxidative stress response, including: glutathione synthase (*p* = 4.5x10^-2^), hydrogen peroxide catabolism (*p* = 3.7x10^-2^), and increased enrichment of antioxidant (*p* = 1.8x10^-2^) and peroxidase activity (*p* = 1.9x10^-2^) (**Figure 3B**). Lastly, several essential defense processes were enriched across all three cultivars including glutathione (*p* = 6.2x10^-75^) and ROS (*p* = 8.4x10^-7^) metabolism, and chitinase activity (*p* = 2.1x10^-18^) (**Figure 3B**).

### Variant identification and expression analysis between FHB-resistant and FHB-susceptible cultivars

**Figure 5:**
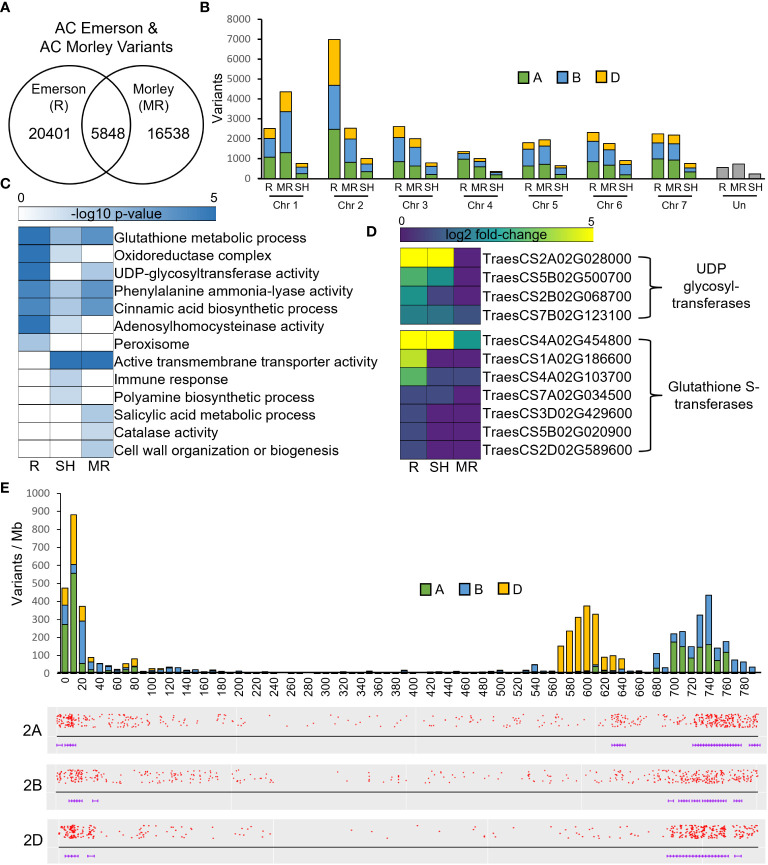
Variant analysis of FHB-resistant AC Emerson (R) and AC Morley (MR) relative to FHB-susceptible CDC Falcon (S). **(A)** Total variants detected within AC Emerson and AC Morley relative to CDC Falcon, including shared variants between AC Emerson and AC Morley. **(B)** Chromosomal diversity of total variants detected. **(C)** GO enrichment of genes containing variants specific to and shared between AC Emerson and AC Morley. **(D)** Expression of UDP-glycosyltransferases and glutathione S-transferases containing variants in AC Emerson relative to CDC Falcon. **(E)** Barplots of variants identified between AC Emerson and CDC Falcon along the 2A, 2B and D chromosomes. SH = shared between AC Emerson and AC Morley. Variants detected using BCFtools software.

**Figure 6:**
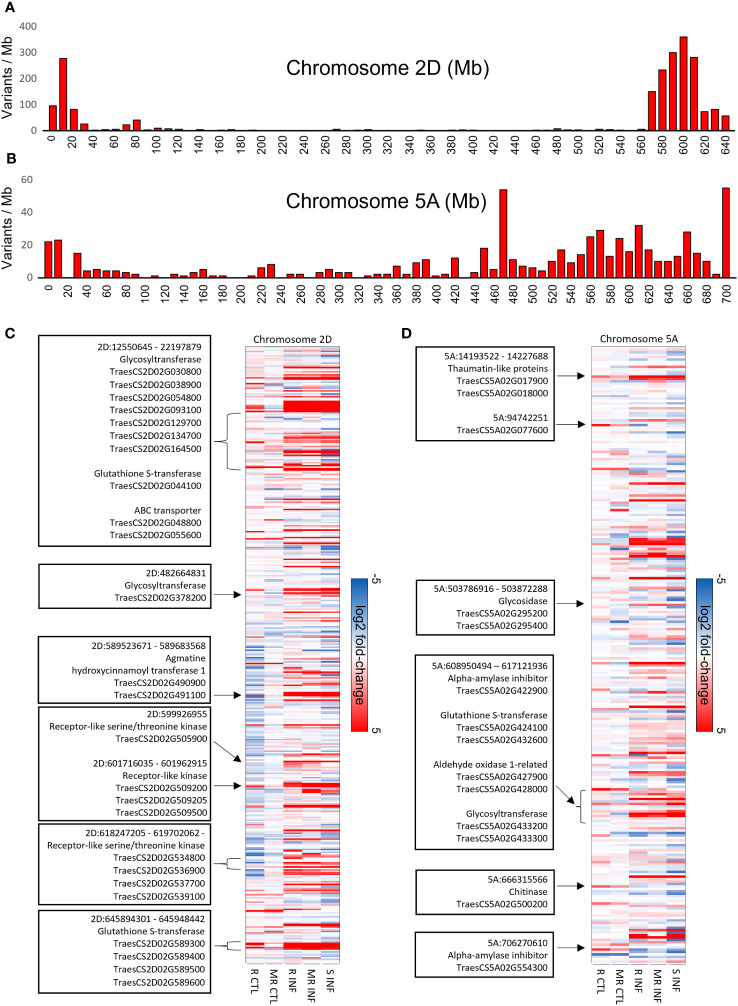
Variant analysis of chromosomes 2D and 5A in FHB-resistant AC Emerson (R) and AC Morley (MR) relative to FHB-susceptible CDC Falcon (S). Barplots of variants identified between AC Emerson and CDC Falcon along the **(A)** 2D and **(B)** 5A chromosomes. Gene expression heatmap of variants identified within gene regions in chromosomes **(C)** 2D and **(D)** 5A. Highlighted genes/regions represent putative defense genes which demonstrate up-regulation in AC Emerson relative to CDC Falcon. Expression values calculated using log_2_ fold change between FHB-resistant cultivars and CDC Falcon for control uninfected tissue, and between uninfected and infected tissues for infected sampels. R = AC Emerson, MR AC Morley and S = CDC Falcon. Variants detected using BCFtools software.

To identify regions of genomic diversity between our FHB-resistant and FHB-susceptible cultivars, we performed a variant analysis comparing AC Emerson and AC Morley against CDC Falcon. Here, we identified 20,401 variants specific to AC Emerson, 16,538 variants specific to AC Morley and 5,848 variants shared between both AC Emerson and AC Morley relative to CDC Falcon (**Figure 5A**). These variants were then plotted across individual chromosomes, where chromosome 2A, B and D contained the highest rate of variants per million bases in AC Emerson with 3.17, 2.75 and 3.53 variants/Mb respectively, while chromosome 1A, B and D showed the highest rate in AC Morley, with 2.20, 2.99 and 2.02 variants/Mb respectively (**Table 1**) (**Figure 5B**). To explore the biological processes and pathways potentially influenced by these variants, we performed a GO enrichment analysis on genes containing these identified variants in both AC Emerson and AC Morley (**Figure 5C**). Similarly to our differential expression analysis, we identified enrichment of putative FHB-resistance related pathways including, UDP-glycosyltransferase activity (*p* = 8.66x10^-6^), lignin biosynthesis through cinnamic acid biosynthesis (*p* = 4.68x10^-5^) and phenylalanine ammonia-lyase activity (*p* = 3.55x10^-5^), and glutathione S-transferases in glutathione metabolism (*p* = 1.89x10^-8^)(**Figure 5C**). Multiple identified UDP-glycosyltransferases and glutathione S-transferases containing variants also demonstrated significant up-regulation in AC Emerson relative to CDC Falcon (**Figure 5D**). Further, we plotted variants identified across the 2A, B and D chromosomes in AC Emerson relative to CDC Falcon where we found clusters of sequence diversity located on both ends of the chromosomes, while maintaining low levels of diversity in-between these distal regions (**Figure 5E**). Here, ~ 90% of variants occurred in the first and last 100 Mb (**Figure 5E**). We also explored variants within coding sequences of chromosomes 2D and 5A specifically to identify regions of nucleotide diversity within putative defense-related genes that also demonstrate differential expression between FHB-resistant and FHB-susceptible cultivars (**Figure 6**). Here, we found several differentially expressed variant containing genes encoding glycosyltransferases, glutathione S-transferases and receptor-like kinases in both chromosomes, as well as thaumatin-like proteins (*TRAESCS5A02G017900*, *TRAESCS5A02G01800*, *TRAESCS5A02G077600*) and α-amylase inhibitors (*TRAESCS5A02G422900*, *TRAESCS5A02G554300*) in chromosome 5A and agmatine hydroxycinnamoyl transferases (TRAESCS2D02G490900, TRAESCS2D02G491100) in chromosome 2D (**Figures 6C, D**).

**Table 1:** Variants identified per chromosome between AC Emerson/CDC Falcon and AC Morley/CDC Falcon.

Chr	Length (bp)	Variants	Variants/Mb	Variants	Variants/Mb
		AC Emerson		AC Morley	
1A	594 102 056	1073	1.81	1305	2.20
1B	689 851 870	945	1.37	2061	2.99
1D	495 453 186	503	1.02	999	2.02
2A	780 798 557	2478	3.17	818	1.05
2B	801 256 715	2205	2.75	1172	1.46
2D	651 852 609	2298	3.53	537	0.82
3A	750 843 639	850	1.13	631	0.84
3B	830 829 764	1211	1.46	950	1.14
3D	615 552 423	552	0.90	421	0.68
4A	744 588 157	970	1.30	585	0.79
4B	673 617 499	296	0.44	281	0.42
4D	509 857 067	93	0.18	134	0.26
5A	709 773 743	635	0.89	726	1.02
5B	713 149 757	841	1.18	913	1.28
5D	566 080 677	330	0.58	315	0.56
6A	618 079 260	849	1.37	676	1.09
6B	720 988 478	1029	1.43	774	1.07
6D	473 592 718	441	0.93	310	0.65
7A	736 706 236	992	1.35	933	1.27
7B	750 620 385	800	1.07	808	1.08
7D	638 686 055	452	0.71	453	0.71
Un	480 980 714	558	1.16	736	1.53

### FHB-resistant cultivars influence mycotoxin biosynthesis and redox homeostasis in *F. graminearum*

**Figure 7:**
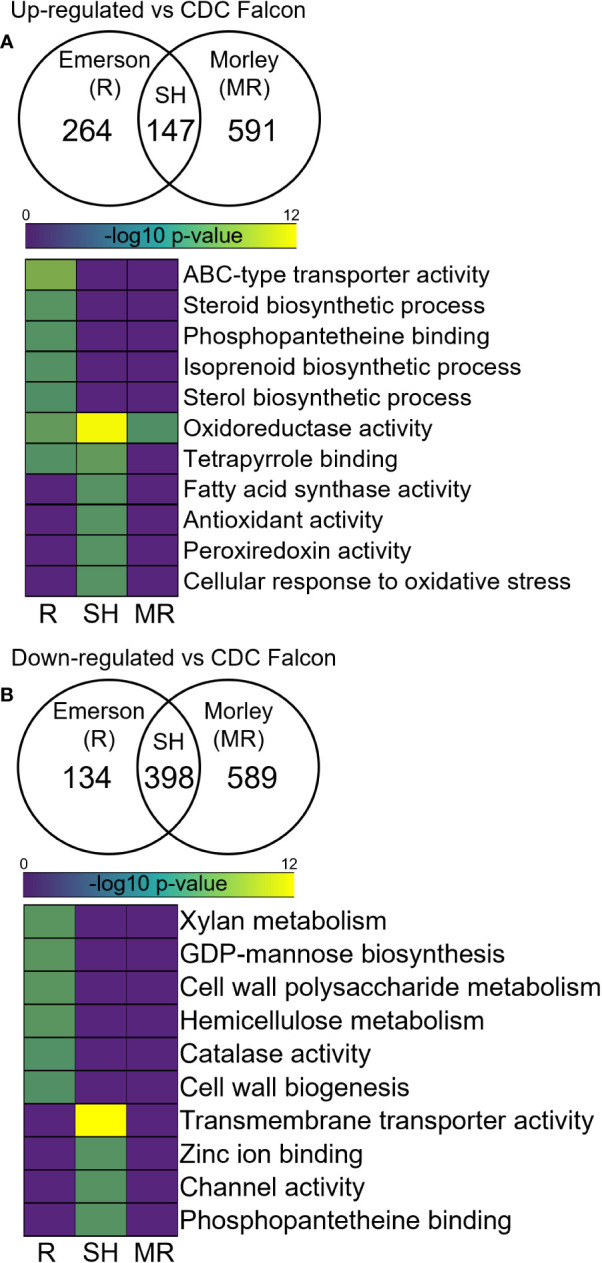
Differential expression analysis in *F. graminearum* infecting AC Emerson (R) and AC Morley (MR) against CDC Falcon (S). Gene ontology term enrichment of significantly **(A)** up-regulated and **(B)** down-regulated genes in *F. graminearum*. DEGs identified using DESeq2 with an adjusted p-value cut-off of *p* < 0.05. GO terms considered significantly enriched at *p* < 0.05 prior to log_10_ transformation. R = FHB-resistant AC Emerson, MR = FHB-moderately resistant AC Morley, SH = shared. Yellow colour = more statistically enriched, purple colour = less statistically enriched.

**Figure 8:**
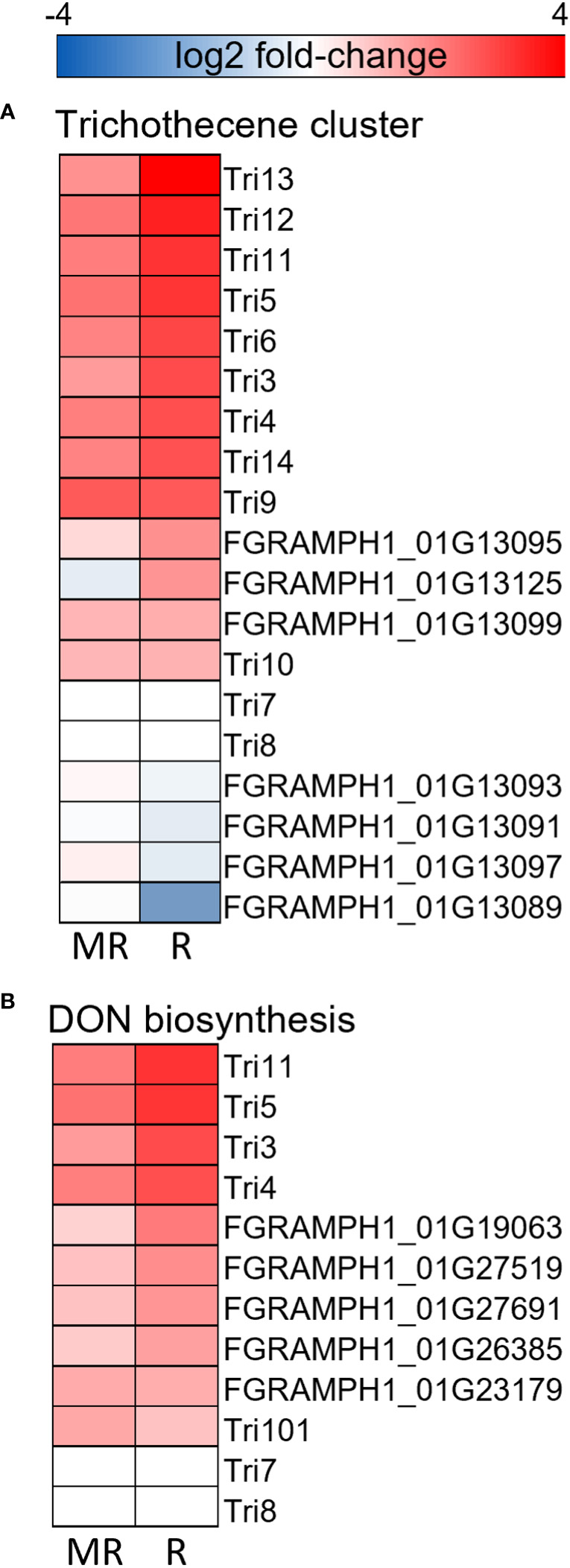
Gene expression heatmaps of the **(A)** trichothecene cluster, **(B)** DON biosynthesis pathway. Heatmaps represent log2 fold-change between AC Emerson (R) and AC Morley (MR) against CDC Falcon (S). Red colour = up-regulated in R, MR relative to S, blue colour = down-regulation in R, MR relative to S.

Next, we compared the RNA originating from *F. graminearum* within infected plants to explore differential expression in *F. graminearum* challenging these resistant and susceptible wheat cultivars to identify genes and processes in the pathogen influenced by distinct defense responses in the host cultivar. Here, we identified 591 and 589 significantly up- and down-regulated genes respectively in *F. graminearum* RNA infecting AC Morley, and 264 and 134 up- and down-regulated genes respectively in *F. graminearum* infecting AC Emerson relative to CDC Falcon (**Figures 7A, B**). Further, we identified 147 and 398 shared up- and down-regulated genes shared between both *F. graminearum* from AC Morley and AC Emerson (**Figures 7A, B**). GO enrichment of up-regulated gene sets showed significant enrichment of ABC-type transporters (*p* = 7.9x10^-5^) associated with DON export, isoprenoid biosynthesis (*p* = 3.2x10^-2^) and phosphopantetheine binding (*p* = 2.4x10^-2^) in *F. graminearum* from AC Emerson (**Figure 7A**). These enriched GO terms contained several up-regulated polyketide synthases belonging to the trichothecene cluster and DON biosynthesis pathway, including *TRI5* and *TRI6* which regulate and are necessary for DON biosynthesis (**Figures 8A, B**)(Cuzick et al., 2008; Wang et al., 2023). Further, several terms associated with the ROS response were enriched in both *F. graminearum* from AC Morley and AC Emerson, including oxidoreductase activity (*p* = 3.2x10^-12^), tetrapyrrole binding (*p* = 3.4x10^-3^), antioxidant activity (*p* = 2.1x10^-2^) and response to oxidative stress (*p* = 2.1x10^-2^) (**Figure 7A**). Meanwhile, *F. graminearum* from AC Emerson-specific down-regulated genes showed enrichment of cell wall biogenesis (*p* = 3.9x10^-2^), xylan metabolism (*p* = 1.2x10^-2^), hemicellulose biosynthesis (*p* = 1.2x10^-2^) and GDP-mannose biosynthesis (*p* = 1.2x10^-2^) (**Figure 7B**).

## Discussion

### Host defense priming in FHB-resistance

As our three winter wheat cultivars demonstrated distinct levels of FHB-resistance (**Figure 1**), we performed a transcriptomic analysis of both uninfected and infected tissues to identify genes and biological processes underlying these different responses. When comparing the three cultivars, differential gene expression and GO analysis of uninfected control tissues identified enriched defense processes in AC Morley and AC Emerson relative to FHB-susceptible CDC Falcon. Enrichment of oxidoreductase activity and cellular redox homeostasis in the shared gene set suggests a potential priming of redox activity prior to infection in these resistant cultivars (**Figure 2C**). This may lead to a more immediate and enhanced response to oxidative stress, which is typically triggered in response to pathogen attack, as ROS are produced and accumulate at the host-pathogen interface upon infection, requiring antioxidant activity to prevent cellular damage (Khaledi et al., 2016). ROS production can be further elicited throughout infection by trichothecenes produced by *F. graminearum*, ultimately resulting in host programmed cell death (Waśkiewicz et al., 2014; Khaledi et al., 2016). The most common antioxidant induced in response to ROS accumulation in wheat are glutathione S-transferases (GSTs) (Buerstmayr et al., 2021). Increased activity of these GSTs has been previously associated with FHB-resistant cultivars in response to infection, with the GST encoded by *Fhb7* conferring resistance to FHB through trichothecene detoxification (Wang et al., 2020; Kumar et al., 2020; Buerstmayr et al., 2021). As we identified 33 putative GSTs enriched in our uninfected tissues, these data suggest a potential priming of GST activity prior to infection that may provide an increased ability to detoxify trichothecenes which are produced by *F. graminearum* during early stages of FHB infection (24 – 96 hpi) (Gunupuru et al., 2017).

AC Emerson-specific enrichment of cutin biosynthesis, as well as chitinase and glucosidase activity were also identified through GO analysis of uninfected control tissues (**Figure 2**). Chitinases and β-glucosidases are commonly up-regulated by hosts immediately upon pathogen recognition to degrade the invading pathogen, where upon secretion, these enzymes can breakdown fungal cell wall components, subsequently acting as signalling molecules to activate host immunity pathways (Wan et al., 2008). In a study by Anand et al., 2003, co-expression of two pathogenesis-related (PR) genes encoding a chitinase and β-1,3-glucosidase led to increased resistance in transgenic spring wheat compared to background FHB-susceptible lines. Further, increased chitinase and glucosidase activity was identified in the FHB-resistant cultivar Sumai 3 (Kosaka et al., 2015). Specific chitinase and glucosidase genes have also been identified in resistance against rust diseases, with the chitinase *CHT20.2* (*TRAESCS5B02G403700*) conferring resistance to wheat stripe rust (Bai et al., 2021) and the β-1,3-glucosidase *TRAESCS3A02G483000* being significantly up-regulated in the yellow stripe rust resistant cultivar Gannong 2 (Zhao et al., 2022). Our data identified both *CHT20.2* and *TRAESCS3A02G483000* as significantly up-regulated in uninfected AC Emerson tissue relative to CDC Falcon (**Dataset S1**). Here, activity of these enzymes, prior to infection, may lead to a more immediate and enhanced pathogen recognition, breakdown and therefore immune signalling response.

Further, cutin is the major component of the outer waxy cuticle which serves as a physical barrier to abiotic and biotic stressors, acting as the first point of contact to pathogen attack (Serrano et al., 2014). Genes responsible for cuticle reinforcement were found to be enriched in FHB-resistant barley, with knockdown of the regulatory transcription factor of these genes (HvWIN1) in RNAi transgenic lines leading to reduced resistance (Kumar et al., 2016). As our data indicates significant enrichment of cutin biosynthesis in FHB-resistant AC Emerson, cuticle density and composition at the point of pathogen contact may be associated with increased tolerance to FHB through slowing pathogen attack and allowing the host sufficient time to mount a successful defense response.

Taken together, these data suggest a priming of the defense response through enhanced antioxidant activity and redox homeostasis shared between resistant cultivars, while AC Emerson further demonstrates amplified enrichment of cellular detoxification through increased glutathione S-transferase activity, as well as enrichment of chitinases and glucosidases capable of degrading fungal cell walls upon infection. These processes, as well as bolstered physical barriers, have historically been essential during early stages of pathogen attack (Kosaka et al., 2015; Wang et al., 2020), which may provide insight into why priming of these responses are associated with FHB-resistance in AC Emerson. Lastly, further transcriptomic investigation of earlier infection stages could help determine whether priming of these host defense pathways results in improved antioxidant activity and pathogen degradation at the onset of FHB infection in AC Emerson and AC Morley cultivars.

### Differential expression of lignin biosynthesis and DON detoxification in FHB-resistant cultivars in response to *F. graminearum*

Differential expression analyses between uninfected and *F. graminearum* infected tissues was subsequently performed to provide insight into the specific genes and pathways that may be critical to FHB resistance during infection. GO analysis of the up-regulated shared AC Morley and AC Emerson gene set in response to infection revealed enrichment of cell wall lignification through chorismate, cinnamic acid and lignin biosynthesis (**Figure 3B**). Cell wall lignification is a common defense response to pathogen attack which bolsters physical defense by filling gaps present between the cellulose, hemicellulose and pectin matrix of plant cell walls (Wang et al., 2013; Ziv et al., 2018). Prior studies have reported increased lignin content as well as increased activation of lignin biosynthesis regulators in FHB-resistant cultivars upon infection relative to susceptible lines (Kang and Buchenauer, 2000; Soni et al., 2021), with genes belonging to the caffeoyl-coenzyme A O-methyltransferase (CCoAOMT) family being identified as key regulators of lignin biosynthesis and therefore potentially FHB-resistance (Yang et al., 2021). Dehydrogenative polymerization of *p*-coumaryl, sinapyl and coniferyl alcohols *via* laccases and class III peroxidases give rise to the *p*-hydroxyphenyl (H), guaiacyl (G) and syringyl (S) units of lignin respectively (Nguyen et al., 2016; Yang et al., 2021). Distinct enzymatic families contribute to the synthesis of these specific monolignol units, with CCoAOMTs being associated with G-unit monolignol synthesis and ferulate-5-hydroxylase (F5Hs) being associated with S-unit synthesis (Cao et al., 2022). Lignin composition, specifically the S/G unit ratio, has been primarily explored for its affect on biomass recalcitrance, however, studies have also demonstrated an influence on disease resistance in plants (Bhuiyan et al., 2009; Cao et al., 2022). A recent study by Cao et al. (2022) demonstrated that silencing of *F5H*, which is necessary for guaiacyl (G) conversion to the syringyl (S) monolignol unit, leads to reduced S/G ratios and increased *S. sclerotiorum* resistance in *f5h Brassica napus* knockout mutants. Interestingly, our data suggests a similar pattern, with FHB-resistant cultivars AC Emerson and AC Morley demonstrating reduced expression of *F5H* genes relative to FHB-susceptible CDC Falcon (**Figure 4A**). Further, we also find increased expression of *CCOAOMT* genes associated with G-unit synthesis in AC Emerson and AC Morley relative to CDC Falcon (**Figure 4A**). These data suggest that lignin composition and the S/G unit ratio of lignin polymers may contribute to resistance against FHB in wheat through the activity of key monolignol synthesis enzymes. Further, the final step of lignin biosynthesis is monolignol polymerization through oxidation of *p*-coumaryl, sinapyl and coniferyl alcohols *via* activity of laccases and class III peroxidases (Nguyen et al., 2016; Rossini et al., 2022). Differential expression analysis identified two plant laccases (*TRAESCS5B02G149900* (*LAC149900*) and *TRAESCS3B02G37990* (*LAC37990*)) to be significantly up-regulated in AC Emerson in response to FHB (**Figure 4A**). Identification of specific laccases and characterization of their role in lignification in response to FHB has yet to be explored in wheat, partly due to the large number of predictive laccases identified in the genome (Hoffmann et al., 2020; Sun et al., 2022). Here, identification of *LAC149900* and *LAC37990* could serve as ideal candidates for further characterization in cell wall lignification in response to FHB.

While laccases are known to play a role in physical defense through their role in lignin polymerization, a study by Sun et al. (2022) also suggested that these laccases can interact with DON, potentially preventing DON from entering host cells through direct interaction or oxidization. Data on laccases and their ability to interact with DON are currently incomplete, however, DON detoxification has primarily been demonstrated through UDP-glycosylation, where DON is conjugated into its detoxified product deoxynivalenol-3-glucoside (Pasquet et al., 2016; Tian et al., 2016). This DON glycosylation *via* UDP-glycosyltransferases has been previously demonstrated to increase resistance against FHB in wheat (Li et al., 2017; He et al., 2020), and within our shared AC Emerson and AC Morley up-regulated gene set, we found significant enrichment of UDP-glycosyltransferase activity (**Figure 3B**). While identified UDP-glycosyltransferases showed significant up-regulation in both AC Morley and AC Emerson cultivars, many genes showed a stronger or specific (*TRAESCS2A02G056400* and *TRAESCS2B02G626500*) up-regulation in AC Emerson relative to CDC Falcon (**Supplementary Figure 4**). As our dataset identified a broader pattern of increased UDP-glycosyltransferase enrichment correlating to increased levels of FHB-resistance, these specific highly up-regulated UDP-glycosyltransferases in resistant cultivars relative to susceptible CDC Falcon may serve as ideal candidate genes for further characterization for their role in DON detoxification and FHB-resistance.

AC Emerson also demonstrated an enrichment of processes associated with oxidative stress, including: hydrogen peroxide catabolism, peroxidase activity, antioxidant activity and glutathione synthase activity (**Figure 3B**). As previously mentioned, the ability to maintain oxidation levels and reduce oxidative stress is critical for stress tolerance in response to both abiotic and biotic stressors (Matić et al., 2021), as ROS can accumulate at high levels requiring both enzymatic and non-enzymatic antioxidants to scavenge the abundant free radicals (Kapoor et al., 2019). Peroxidases are multi-functional enzymes, demonstrating antioxidant activity through hydrogen peroxide catabolism in response to stress (Tyagi et al., 2018), while also contributing to lignin polymerization in cell wall reinforcement through class III peroxidases (Hoffmann et al., 2020). ROS can also be detoxified through non-enzymatic antioxidants like glutathione, whose synthesis is catalyzed *via* glutathione synthase (Hasanuzzaman et al., 2017). Increased levels of glutathione during early infection stages has been previously associated with increased levels of tolerance across multiple plant pathosystems (Dubreuil-Maurizi and Poinssot, 2012; Zechmann, 2020). Further, glutathione and ROS metabolism were also enriched across all three cultivars in response to FHB, along with increased chitinase activity (**Figure 3B**). Interestingly, while enrichment of these processes were shared across all three cultivars in response to infection, they were also enriched specifically within uninfected AC Emerson tissue compared uninfected AC Morley and CDC Falcon, further suggesting a potential priming of these key defense processes that are critical in response to FHB.

### Variant detection within FHB-resistant cultivars and differential expression analysis of sequence diverse regions

QTLs associated with FHB-resistance have predominately been identified in spring wheat cultivars, with the most well established and repeatable QTLs occurring on chromosomes 3B (*Fhb1; Qfhs.ndsu-3BS*), 5A (*Fhb5*; *Qfhs.ifa-5A*) and 6B (*Fhb2*). However, a study by Clinesmith et al., 2019 identified four QTLs conferring resistance to FHB in winter wheat cultivars, with two of the four being located on chromosome 2D. Further, one of these QTLs (*Qfhb.ksu-2D.1*) was identified at 580.12 Mb, a region where we identified high levels of variation in AC Emerson (**Figure 5G**). Two FHB disease index marker trait associations (MTAs) were also identified on chromosome 2A and 2B in winter wheat, where similarly to *Qfhb.ksu-2D.1*, these MTAs were located towards the end of the long arm of chromosomes 2A and 2B, occurring at the 722 and 725 Mb positions respectively (Zhang et al., 2022). These chromosomal regions also demonstrate high levels of sequence diversity in AC Emerson (**Figures 5E, F**), suggesting these regions may be contributing to FHB resistance across multiple winter wheat cultivars. The majority of variants identified in both AC Emerson and AC Morley occurred at the distal regions of chromosomes (**Dataset S4**) (**Figures 5E-G**). In a study by Nilsen et al. (2021) exploring variants present in chromosome 5A, a similar phenomena was discovered, with a significant number of variant clusters occurring near the end of chromosome 5A. This disproportionate clustering of variants is likely due to the increased recombination rates associated with the distal regions of chromosomes in crop species with large genomes, including wheat, maize and barley (Ganal et al., 2011; Mayer et al., 2012; Choulet et al., 2014). Further, while these distal chromosomal regions have demonstrated greater gene density across several grass genomes (Schnable et al., 2009; Choulet et al., 2014), GO analysis has also described an enrichment of genes associated host stress responses in these distal genes (Choulet et al., 2014). These data suggest distal chromosomal regions may be of particular interest in the discovery of FHB and other resistance associated QTLs. GO enrichment of variants within annotated genes in our data identified high levels of enrichment for similar processes and pathways as identified in our differential expression analysis, including UDP-glycosyltransferase activity, glutathione S-transferase activity and lignin biosynthesis through phenylalanine ammonia-lyase activity and cinnamic acid biosynthesis (**Figure 5C**). These data suggest that sequence variation in genes associated with these processes may contribute to their differential expression across AC Emerson and AC Morley cultivars relative to CDC Falcon.

To further explore these findings we mapped expression of all gene variants identified in chromosomes 2D and 5A, as multiple important FHB-related QTLs have been previously identified in these regions (McCartney et al., 2016; Clinesmith et al., 2019; Steiner et al., 2019). Here, we discovered two distinct clusters of differentially expressed receptor-like kinases (RLKs), known for their role in defense signalling, within chromosome 2D located at the 601 and 618 Mb regions (**Figure 6C**). A previous study by Hu et al., 2019 identified four receptor-like kinases as DEG candidates genes associated with the FHB 2DL-QTL, as these RLKs showed significant up-regulation in FHB-resistant near isogenic lines. While several of the RLKs identified in our study were induced in response to *F. graminearum*, multiple were also upregulated in uninfected AC Emerson relative to CDC Falcon coupled with a stronger induction in response to infection (**Figures 6C, D**). This increased activity prior to infection may result in a more immediate signalling response and subsequent down-stream defense gene activation upon infection. We also identified variants within two agmatine hydroxycinnamoyl transferases (*TRAESCS2D02G490900*, *TRAESCS2D02G491100*) that demonstrated up-regulation in response to *F. graminearum* in AC Emerson. Hydroxycinnamoyl transferases are involved in lignin biosynthesis through the synthesis of hydroxycinnamic acid amides (HCAAs), where silencing of these transferases has been shown to inhibit lignin polymerization in *N. benthamiana* (Hoffmann et al., 2004). The agmatine coumaroyl transferase, *TaACT*, has previously been described in the FHB QTL-2DL and is a rate limiting step in the synthesis of HCAAs, where silencing of *TaACT* leads to reduced HCAA accumulation and increased FHB susceptibility (Kage et al., 2017). As hydroxycinnamoyl transferases also play a vital role in HCAA production, *TRAESCS2D02G490900* and *TRAESCS2D02G491100* may also be associated with FHB resistance on chromosome 2D through HCAA biosynthesis. Further, several differentially expressed glycosyltransferases and glutathione S-transferases were once again identified across both chromosomes 2D and 5A, however, on chromosome 5A we also found thaumatin-like proteins (TLPs) and α-amylase inhibitors which were significantly up-regulated in AC Emerson relative to CDC Falcon prior to infection (**Figures 6C, D**). Thaumatin-like proteins belong to the pathogenesis-related (PR) protein 5 family and are commonly induced in response to fungal pathogen attack (Liu et al., 2010; Ren et al., 2022). TLPs have demonstrated the ability to inhibit pathogen growth across several plant species as well as *in vitro* (Ren et al., 2022). A previous genome wide characterization of thaumatin-like proteins in response to FHB infection identified a disproportionate distribution of these genes across the genome, with the highest number of TLPs (22) being found on chromosome 5A (Ren et al., 2022). A further analysis of differential expression between *F. graminearum* inoculated and mock inoculated near isogenic lines expressing *Fhb1* and *Qfhs.ifa-5A* FHB-resistance QTLs showed *TRAESCS5A02G017900* to be one of four TLPs to be significantly up-regulated across all timepoints tested (24hpi – 48 hpi) (Ren et al., 2022). Our data indicates not only do we find induction of this TLP in response to *F. graminearum* at 7 dpi across AC Emerson and AC Morley, we also find increased activity in AC Emerson relative to CDC Falcon prior to infection (**Figure 6D**). Further, variants in two α-amylase inhibitors were identified on chromosome 5A that also demonstrate significant up-regulation in AC Emerson relative to CDC Falcon prior to infection (**Figure 6D**). These α-amylase inhibitors have been shown to accumulate at significantly higher levels in wheat kernels of FHB-resistant cultivars relative to FHB-susceptible lines (Perlikowski et al., 2014). α-amylases are necessary for starch hydrolysis in *Fusarium graminearum*, where reducing α-amylase activity can lead to reduced trichothecene production and ultimately reduced pathogenicity in wheat (Oh et al., 2016) Further, these α-amylase inhibitors have been shown to inhibit pathogen growth *in vitro* and reduce mycotoxin production by the pathogen (Mendes et al., 2015).

### FHB-resistant cultivars influence mycotoxin biosynthesis and redox homeostasis in *F. graminearum*

Differential expression analysis of *F. graminearum* infecting AC Emerson, AC Morley and CDC Falcon revealed significant up-regulation of genes in mycotoxin clusters and biosynthesis pathways in FHB-resistant cultivars (**Figure 8**). Trichothecene mycotoxins produced by *Fusarium* species have been demonstrated to increase pathogenicity in multiple hosts, acting through inhibition of protein synthesis and the generation of free radicals leading to oxidative stress (Wu et al., 2017; Villafana et al., 2019). The C2H2 transcription factor *TRI6* has been described as a positive regulator of trichothecene gene expression necessary for DON biosynthesis, with *tri6* mutants demonstrating reduced expression of all *TRI* cluster genes, with the exception of *TRI10* which is also hypothesized to be a positive regulator of *TRI* expression (Wang et al., 2023). Further, *TRI5* activity has also been described as essential for DON biosynthesis, with *tri5* mutants not being able to produce DON and demonstrating significantly reduced pathogenicity (Cuzick et al., 2008). All three positive regulators of *TRI* gene cluster activity and DON biosynthesis (*TRI6*, *TRI10* and *TRI5*) showed significant up-regulation when *F. graminearum* was analyzed from both AC Morley and AC Emerson cultivars, along with several other members of the trichothecene cluster and DON biosynthesis pathway (**Figures 8A, B**). Enrichment of ATP-binding cassette (ABC)-type transporters in *F. graminearum* infected FHB-resistant AC Emerson was also identified (**Figure 7**). ABC-type transporters and major facilitator superfamily (MFS) transporters have been hypothesized to be responsible for the export of DON and other mycotoxins produced by *F. graminearum* during pathogenesis (O’Mara et al., 2021). Prior studies have demonstrated that silencing of *ABC1*, *ABC3* and *ABC6* each result in significantly reduced DON accumulation (O’Mara et al., 2021), while the MFS transporter, *TRI12*, also results in reduced DON accumulation when silenced (Menke et al., 2012). Our data identified *ABC1*, *ABC3*, and *ABC6* to be significantly up-regulated in *F. graminearum* infecting AC Emerson, along with several other ABC-type transporters, while *TRI12* was significantly up-regulated in *F. graminearum* infecting both AC Morley and AC Emerson (**Supplementary Figure 5**; **Dataset S1**). Prior studies have hypothesized that *ABC6* and *MFS1* may be regulated by *TRI6* and play a role in DON export only when *TRI12* is absent, as these transporters were highly up-regulated with *TRI6* in *tri12* mutants during periods of DON production (O’Mara et al., 2021). While our data supported this hypothesis for *MFS1*, which showed no activity in *F. graminearum* infecting AC Emerson or AC Morley cultivars when *TRI12* was up-regulated, *ABC6* did show up-regulation in *F. graminearum* infecting AC Emerson, suggesting these two transporters may not be redundant in function (**Dataset S1**). As export of DON and other mycotoxins has yet to be well characterized, the ABC-type and MFS transporters identified in this study may serve as ideal candidates for further characterization for their role in *F. graminearum* pathogenesis (**Supplementary Figure 5**). Enrichment of these genes involved in the trichothecene cluster, as well as DON biosynthesis and export, could ultimately be due to the suppression at earlier stages of infection in resistant cultivars, leading to a need to up-regulate these key pathogenicity factors. Alternatively, as DON biosynthesis genes and the trichothecene cluster has previously demonstrated increased activity during early stages of infection (48h – 96h) and decreased activity at later stages, these findings could be attributed to the progression of FHB in CDC Falcon and the inability of infection to progress in resistant AC Emerson and AC Morley cultivars beyond the early infection stages where activity of these genes is highest (Lysøe et al., 2011).

Further, oxidative stress has been demonstrated to activate toxin production, specifically the trichothecene cluster where increased H_2_O_2_ leads to increased *TRI* gene expression in *F. graminearum* (Montibus et al., 2013). This correlates with our data, which showed enrichment of the trichothecene cluster and ROS response in *F. graminearum* infecting AC Emerson (**Figures 7A**, **8**). Further, the ROS-degrading enzymes, catalases, have been identified as pathogenicity factors that can neutralize host-derived ROS present at the host-pathogen interface, however, *CAT1* and *CAT3* which have been previously identified as essential in maintaining redox homeostasis, were down-regulated in *F. graminearum* infecting our resistant cultivars relative to infecting the FHB-susceptible cultivar CDC Falcon (Montibus et al., 2016). These data further support a study by Ponts et al. (2007), where catalase treated *F. graminearum* resulted in reduced *TRI* gene expression and toxin accumulation, which is what our data indicates in *F. graminearum* infecting susceptible CDC Falcon where *CAT1* and *CAT3* are expressed. Conversely, down-regulated genes in *F. graminearum* challenging our resistant cultivars demonstrated increased cell wall modification and biogenesis (**Figure 7**). This enrichment suggests reduced fungal growth in resistant cultivars compared to *F. graminearum* in CDC Falcon which is demonstrating consistent growth as indicated by the significantly greater spread of infection and FHB-severity in CDC Falcon.

Taken together, we identified several enriched defense processes prior to and in response to *F. graminearum* infection associated with FHB-resistance in AC Emerson and AC Morley winter wheat cultivars, including: redox homeostasis, lignin biosynthesis and DON detoxification. Within these defense processes we have identified novel putative defense regulators as well as defense genes previously associated with FHB-resistance in other FHB-resistant cultivars and species. Finally, dual RNA-sequencing identified novel putative pathogenicity factors in *F. graminearum* which were differentially expressed when infecting these FHB-resistant cultivars compared to CDC Falcon. These data provide insight into identifying new sources of FHB-resistance in winter wheat and improving our understanding of this important pathosystem.

## Data availability statement

The datasets presented in this study can be found in online repositories. The names of the repository/repositories and accession number(s) can be found in the article/**Supplementary Material**.

## Author contributions

PW: Conceptualization, Data curation, Formal analysis, Investigation, Methodology, Visualization, Writing – original draft. MB: Supervision, Writing – review & editing. BM: Resources, Writing – review & editing. CM: Resources, Writing – review & editing. HR: Resources, Writing – review & editing. MH: Conceptualization, Formal analysis, Funding acquisition, Methodology, Project administration, Resources, Supervision, Writing – review & editing.

## Supplementary material

Supplementary Figure 1 Hierarchical clustering analysis of CDC Falcon, AC Morley and AC Emerson *T. aestivum* control and *F. graminearum* infected (7-dpi) samples. Count data was generated using featureCounts and used as input. Dendrogram generated using the pvclust package in r. CTL, control water inoculated samples; INF, *F. graminearum* infected samples at 7-dpi. A = bio-replicate #1, B = bio-replicate #2, C = bio-replicate #3.

Supplementary Figure 2 Principal component analysis of **(A)** *T. aestivum* (CDC Falcon (S), AC Morley (MR) and AC Emerson (R)) and **(B)** *F. graminearum* infecting CDC Falcon (S), AC Morley (MR) and AC Emerson (R) at 7-dpi. Count data generated using featureCounts was used as input and PCA plot generated using DESeq2 in r. CTL = control water inoculated samples, INF = *F. graminearum* infected samples at 7-dpi.

Supplementary Figure 3 Target *T. aestivum* and *F. graminearum* gene expression correlation of RNA sequencing and qPCR data. Log2 fold-change of AC Emerson and AC Morley cultivars compared to CDC Falcon and deltaCT qPCR expression data. *T. aestivum* expression data comparisons of **(A)** AC Morley vs CDC Falcon and **(B)** AC Emerson vs CDC Falcon. *F. graminearum* expression data comparisons of **(C)** AC Morley vs CDC Falcon and **(D)** AC Emerson vs CDC Falcon. RNA sequencing data represented as bars and qPCR data represented with diamond marked line plot.

Supplementary Figure 4 Gene expression heatmap of UDP-glycosyltransferases. Heatmaps represent log2 fold-change between infected and control tissues for AC Emerson (R), AC Morley (MR) and CDC Falcon (S) cultivars. Red colour = up-regulated infected tissue relative to uninfected, blue colour = down-regulation in infected tissue relative to uninfected.

Supplementary Figure 5 Gene expression heatmap of ABC-type and MFS transporters. Heatmaps represent log2 fold-change between AC Emerson (R) and AC Morley (MR) against CDC Falcon (S). Red colour = up-regulated in R, MR relative to S, blue colour = down-regulation in R, MR relative to S.
