# Supplementary figures and images for: Dual RNA-sequencing of Fusarium head blight resistance in winter wheat
Source: Front Plant Sci. 2024 Jan 4;14:1299461. doi: 10.3389/fpls.2023.1299461 (PMC10794533; doi:10.3389/fpls.2023.1299461)

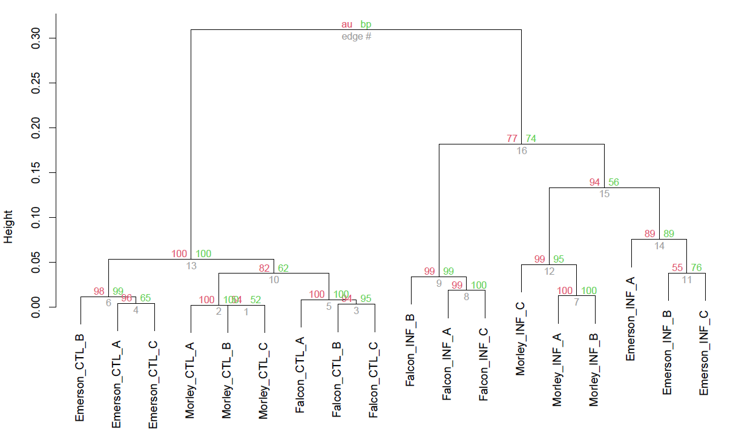

Supplement: Supplementary Figure 1 — Hierarchical clustering analysis of CDC Falcon, AC Morley and AC Emerson T. aestivum control and F. graminearum infected (7-dpi) samples. Count data was generated using featureCounts and used as input. Dendrogram generated using the pvclust package in r. CTL, control water inoculated samples; INF, F. graminearum infected samples at 7-dpi. A = bio-replicate #1, B = bio-replicate #2, C = bio-replicate #3. [file Image_1.tif]

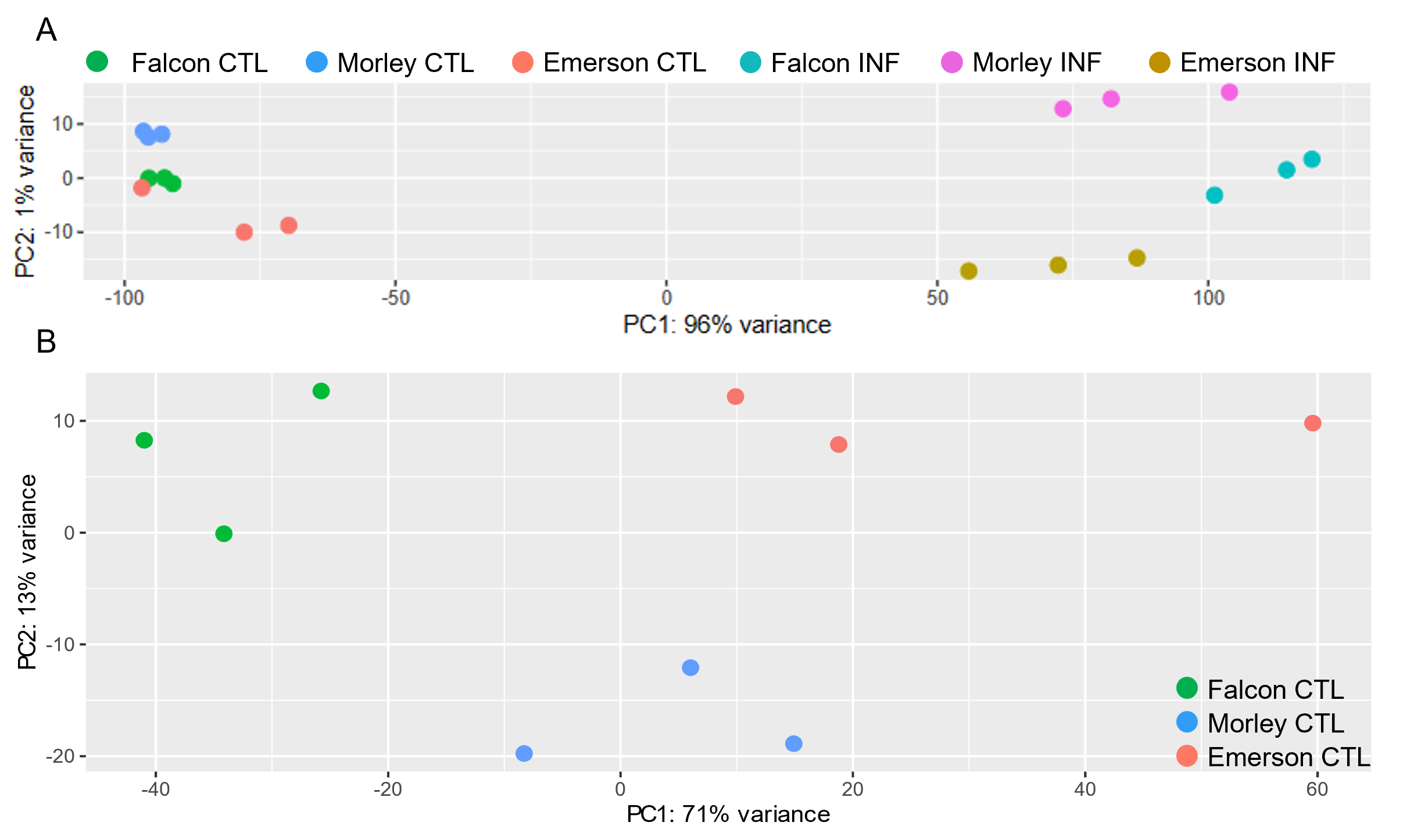

Supplement: Supplementary Figure 2 — Principal component analysis of (A) T. aestivum (CDC Falcon (S), AC Morley (MR) and AC Emerson (R)) and (B) F. graminearum infecting CDC Falcon (S), AC Morley (MR) and AC Emerson (R) at 7-dpi. Count data generated using featureCounts was used as input and PCA plot generated using DESeq2 in r. CTL = control water inoculated samples, INF = F. graminearum infected samples at 7-dpi. [file Image_2.tif]

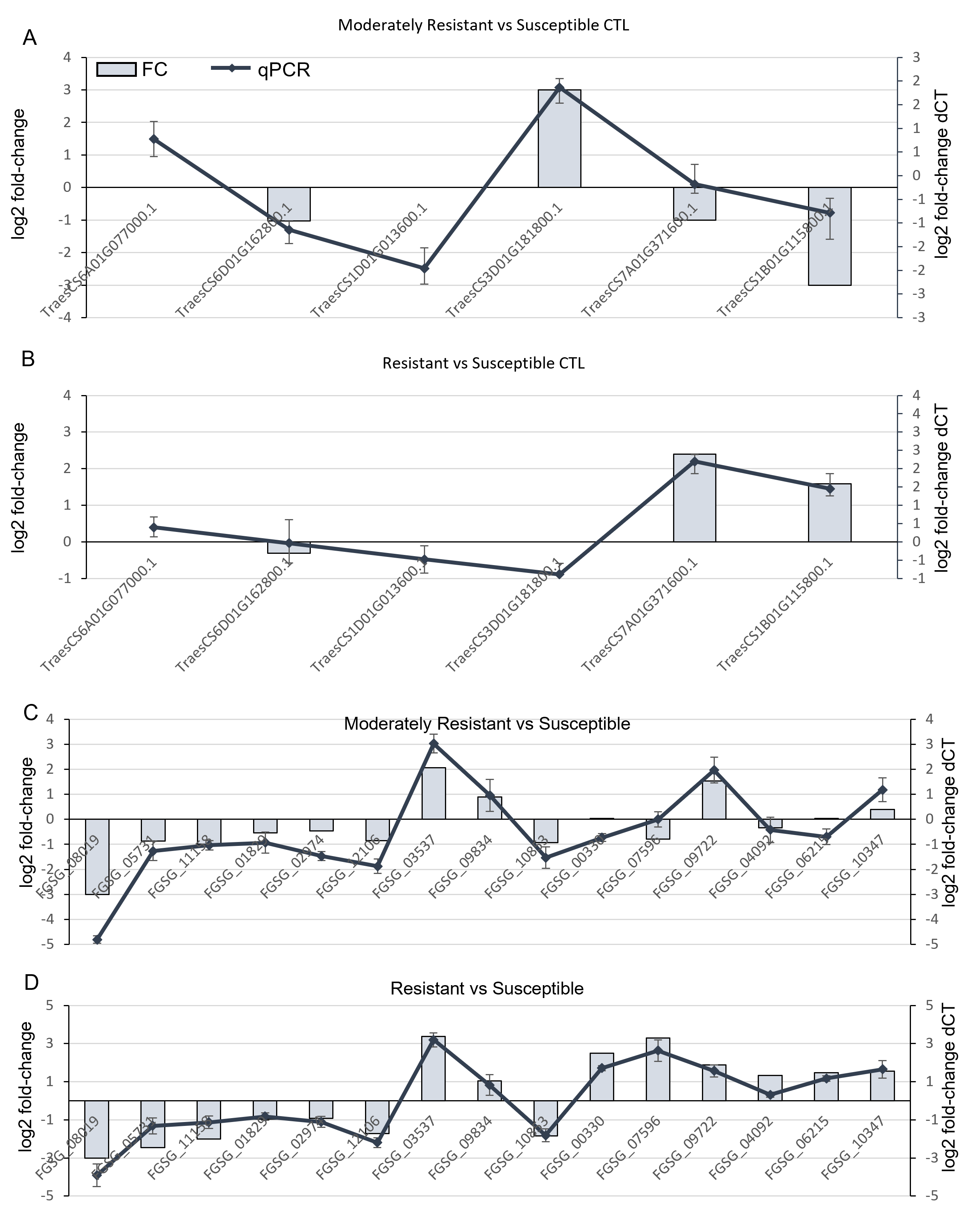

Supplement: Supplementary Figure 3 — Target T. aestivum and F. graminearum gene expression correlation of RNA sequencing and qPCR data. Log2 fold-change of AC Emerson and AC Morley cultivars compared to CDC Falcon and deltaCT qPCR expression data. T. aestivum expression data comparisons of (A) AC Morley vs CDC Falcon and (B) AC Emerson vs CDC Falcon. F. graminearum expression data comparisons of (C) AC Morley vs CDC Falcon and (D) AC Emerson vs CDC Falcon. RNA sequencing data represented as bars and qPCR data represented with diamond marked line plot. [file Image_3.tif]

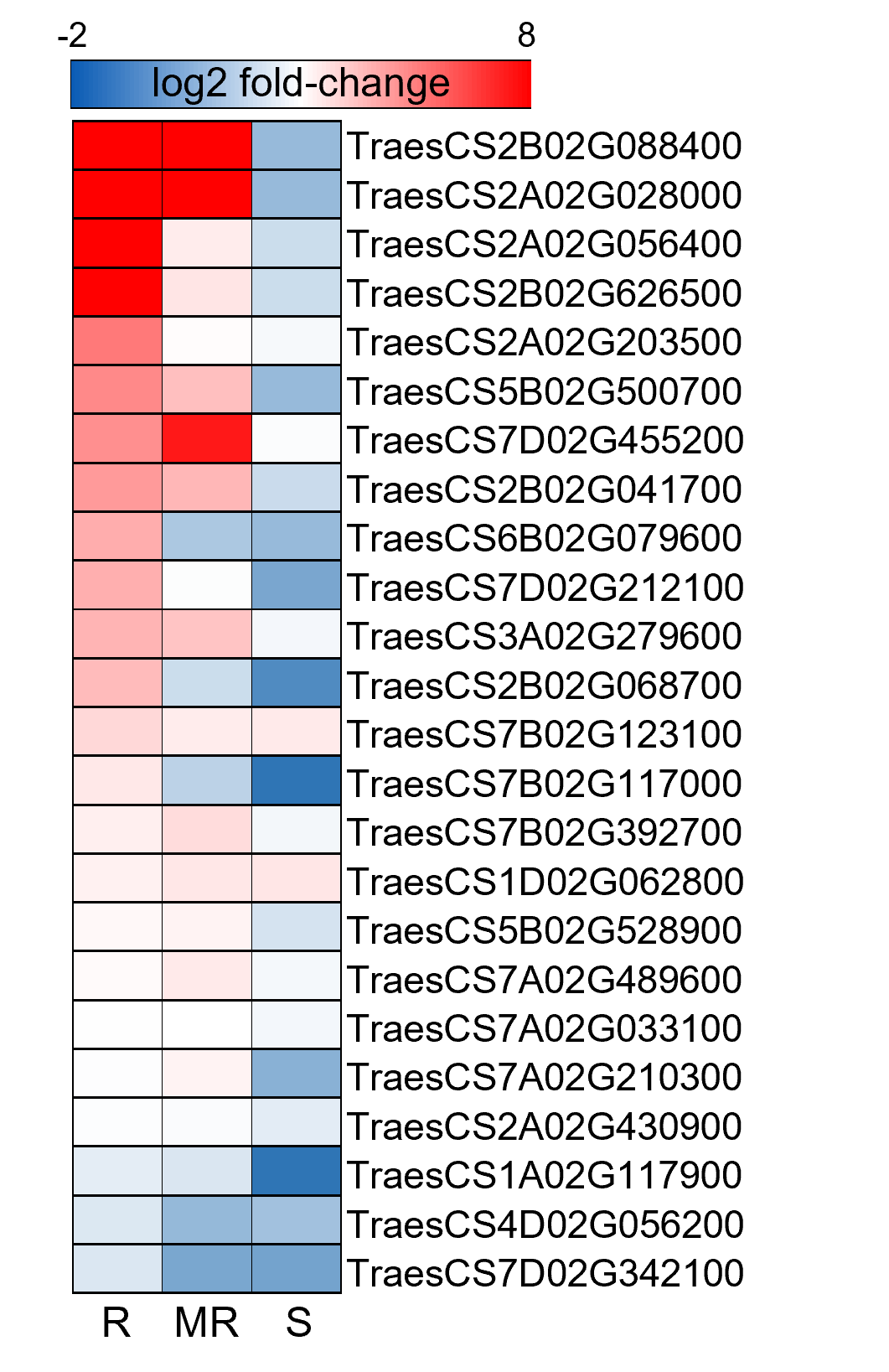

Supplement: Supplementary Figure 4 — Gene expression heatmap of UDP-glycosyltransferases. Heatmaps represent log2 fold-change between infected and control tissues for AC Emerson (R), AC Morley (MR) and CDC Falcon (S) cultivars. Red colour = up-regulated infected tissue relative to uninfected, blue colour = down-regulation in infected tissue relative to uninfected. [file Image_4.tif]

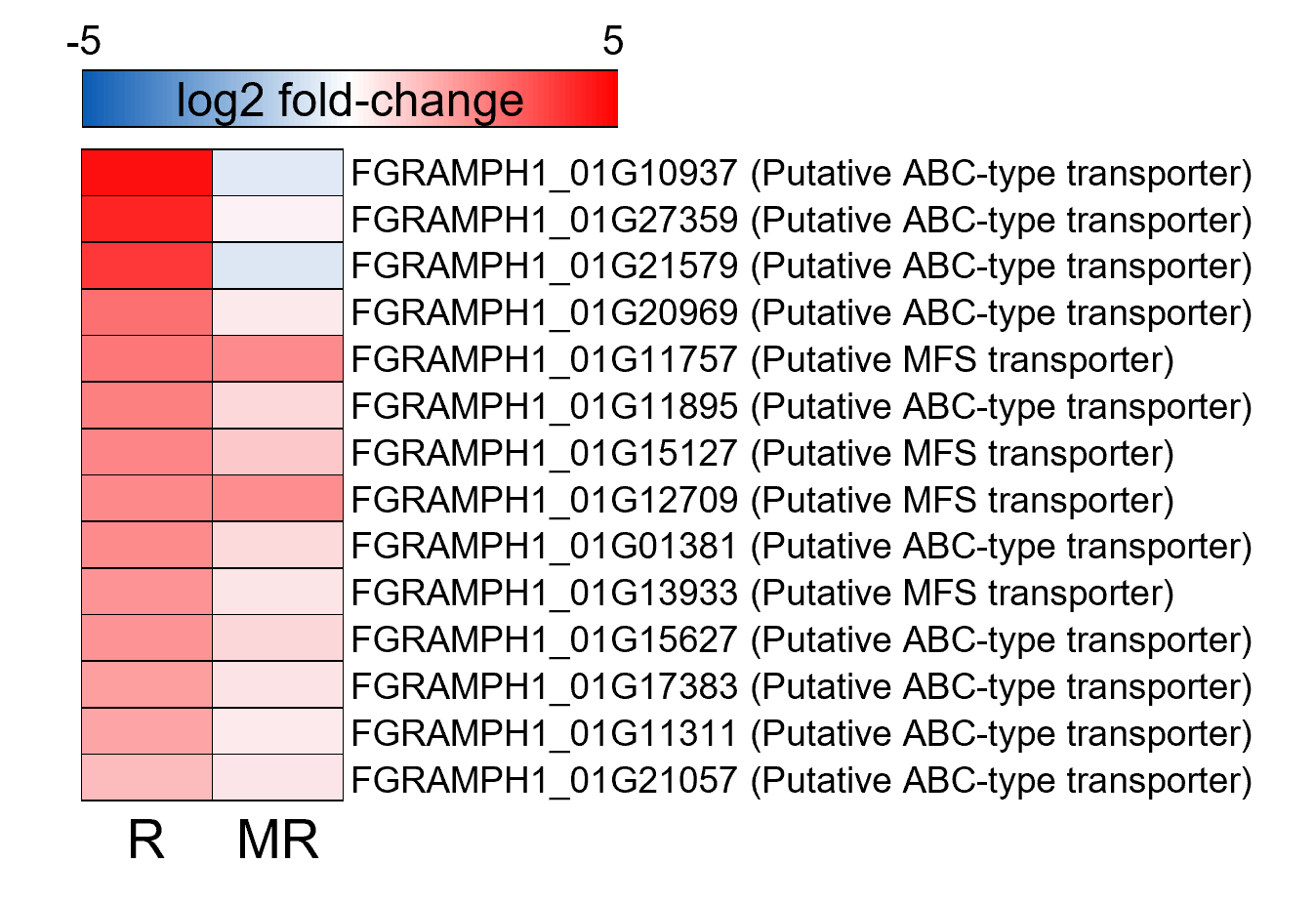

Supplement: Supplementary Figure 5 — Gene expression heatmap of ABC-type and MFS transporters. Heatmaps represent log2 fold-change between AC Emerson (R) and AC Morley (MR) against CDC Falcon (S). Red colour = up-regulated in R, MR relative to S, blue colour = down-regulation in R, MR relative to S. [file Image_5.tif]
